# The Use of Janus Kinase Inhibitors for Lichen Planus: An Evidence-Based Review

**DOI:** 10.1177/12034754231156100

**Published:** 2023-02-23

**Authors:** Abrahim Abduelmula, Ahmed Bagit, Asfandyar Mufti, Katie C.Y. Yeung, Jensen Yeung

**Affiliations:** 1 6221 Faculty of Medicine, University of Western Ontario, London, Ontario, Canada; 2 7938 Faculty of Medicine, University of Toronto, Toronto, Ontario, Canada; 3 12366 Division of Dermatology, Department of Medicine, University of Toronto, Ontario, Canada; 4 104820 Faculty of Medicine, Queen’s University, Kingston, Ontario, Canada; 5 Women’s College Research Institute, Women’s College Hospital, Toronto, Ontario, Canada; 6 7938 Sunnybrook Health Sciences Centre, Toronto, Ontario, Canada; 7 7938 Probity Medical Research, Waterloo, Ontario, Canada

**Keywords:** lichen planus, JAK inhibitor, therapeutics, systemic, systematic review, evidence-based

## Abstract

**Background:**

Lichen Planus (LP) is a dermatological disorder characterized by violaceous papules that affect the cutaneous region, nails, scalp, and mucous membranes. Current molecular and clinical studies point to the Janus Kinase-signal transducer and activator of transcription (JAK-STAT) pathway as a potential effector of LP pathology.

**Objective:**

This systematic review summarizes the current reported literature outcomes for patients receiving JAK inhibitors to treat LP.

**Methods:**

MEDLINE and Embase were searched on 16 October, 2022, and 15 original articles were included, with 56 LP patients.

**Results:**

(mean age: 54.5 years, range: 26-81 years, male: 26.8%). The treatment outcomes were included for the following JAK inhibitors: tofacitinib (*n* = 30), baricitinib (*n* = 16), ruxolitinib (*n* = 12), and upadacitinib (*n* = 2). Patient outcomes were classified into complete resolution, partial resolution, and no resolution. Patients achieving complete resolution represented 25% (*n* = 4/16) in the baricitinib group, 10% (*n* = 3/30) in the tofacitinib group, 16.7% (*n* = 2/12) in the ruxolitinib group, and 100% (2/2) in the upadacitinib group. Partial resolution patients represented 31.3% (*n* = 5/16) of baricitinib patients, 60% (*n* = 18/30) of tofacitinib patients, and 83% (*n* = 10/12) of ruxolitinib patients. 43.8% (*n* = 7/16) of baricitinib patients and 10% (*n* = 9/30) of tofacitinib patients had no resolution of lesions.

**Conclusion:**

This review also highlights the significance of utilizing a uniform outcome measure for LP, as it aids in reporting more generalizable results, reduces reporting bias, and ultimately lead to improved clinical outcomes for LP patients.

## Introduction

Lichen Planus (LP) is a papulosquamous dermatological condition that is typically defined by the presence of pruritic violaceous papules.^1^ LP is most commonly found on the extremities, and the majority of the patient population impacted are middle-aged adults. Although this condition is typically self-limited for range of several months to years, a considerable proportion of the patients are affected indefinitely.^2^ LP typically affects the skin, mucous membranes, scalp, and nails. The symptoms associated with LP tend to be relative to the affected anatomical site, with cutaneous LP presenting with pain and burning symptoms, whereas mucosal LP presents with dysphagia, hoarseness, and stridor.^1^

The pathogenesis of LP is not fully understood yet, however, current literature suggests that the presentation of LP is a consequence of an autoimmune reaction that targets the basal keratinocytes.^3^ There can be a wide array of triggers for LP including, but not limited to, trauma, drugs, contact allergens, and autoimmune diseases. Furthermore, some pathogenic conditions, such as Hepatitis C, have been identified as risk factors that could increase the likelihood of the development of LP.^4^ As it stands, there is no definitive cure for LP, given its wide variety of presentations and causative factors. However, recently published studies have indicated promise in treating LP through targeting the Janus Kinase-signal transducer and activator of transcription (JAK-STAT) pathway.^5^ It has been theorized that cytokines activated by the JAK-STAT pathway are major contributors to the pathogenesis of LP.^6^ As such, inhibiting this pathway could potentially yield positive outcomes, particularly in patients with persistent LP.

In this systematic review, we investigate the impact of JAK-STAT inhibitors on the clinical outcomes of LP patients, through examining previously published studies in the literature. The results of this review will be of benefit to clinical practitioners, who are managing patients with LP.

## Methods

### Search Strategy

This systematic review’s study protocol was designed according to the Preferred Reporting Items for Systematic Reviews and Meta-Analysis (PRISMA) guidelines. A literature search was conducted using MEDLINE and Embase on October 16th, 2022. The term ‘lichen’ was searched along with the variations of JAK inhibitors, which yielded 171 articles (Supplemental File 1).

## Eligibility Criteria

Original articles written in the English language were included if they:

1. reported intervention of interest (i.e., patients on JAK inhibitor therapy),
2. involved study population (i.e., human participants with LP),
3. had an observational (i.e., case reports, case series, cross-sectional or cohort studies) study design.

Studies that did not report on treatment outcomes were excluded. Additionally, conference abstracts and studies with irretrievable full texts were excluded (Supplemental Figure 1).

## Study Selection

The articles obtained from the literature search were screened independently by two reviewers (A.A and K.Y) to verify their eligibility to be included. Any conflicts between the two reviewers were resolved through consulting a third reviewer (A.M). The reference lists of relevant articles were also checked manually to identify studies that were not originally included using the outlined search strategy.

## Data Extraction

Two reviewers extracted data from the included articles using a structured format. Conflicts were discussed among the two reviewers and consultation with a third reviewer was conducted if a resolution was not met. The data extracted included the study, patient characteristics and demographics, LP information, JAK information, and treatment outcomes.

## Level of Evidence

The level of evidence of the included articles was evaluated by two independent reviewers, using The Oxford Centre for Evidence-Based Medicine 2011 Levels of Evidence.^7^ Due to the data’s heterogeneity, only a descriptive analysis was conducted.

## Results

### Study and Patient Characteristics

A total of 171 articles were identified through the literature search strategy. After the screening process was conducted to verify the eligibility criteria, 15 articles were included for full-text review and data collection. The included studies were retrospective reviews (2/15), case reports (10/15), prospective reviews (2/15), and case series (1/15). The studies included a total of 56 patients (mean age: 54.5 years, range: 26-81 years). Males made up 26.8% (15/56) of the patients, and females represented 73.2% (41/56) of the patients. Across the 56 patients, 60 instances of JAK inhibitor use was reported. The JAK inhibitors reported included in the studies were, tofacitinib (50%, 24/60), baricitinib (26.7%, 16/60), ruxolitinib (20%, 12/60), and upadacitinib (3.3%, 2/60). LP was noted in 61.7% (37/60) and lichen planopilaris (LPP) in 55% (33/60). The distribution of LP among the cases was: scalp (42.9%, 24/56), forehead (25%, 14/56), classic cutaneous LP (22.2%, 12/56), oral mucosa (10.7%, 6/56), nail (3.6%, 2/56), esophageal (3.6%, 2/56), ocular (1.8%, 1/56). Concurrent use of systemic non-JAK inhibitor therapies was noted in 50% (28/56) of patients (Supplemental File 2). Prior systemic non-JAK inhibitor therapies included: topical corticosteroids (62.5%), hydroxychloroquine (41.1%), calcineurin inhibitors (26.8%), systemic corticosteroids (21.4%), and methotrexate (16.1%). Treatment-related adverse events (AEs) were reported in 7 patients (7/56, 12.5%), With baricitinib and tofacitinib resulting in AEs in 3 patients each, and ruxolitinib resulting in AEs in 1 patient.(Supplemental File 3).

### Outcome Measures

The outcome measures compiled from the included studies are summarized in Table 1. Two of the studies (2/15) included assessed their patient cohorts (20/56) based on the Lichen Planopilaris Activity Index (LPPAI), a validated measure of LPP disease severity which numerically quantifies the signs and symptoms of LPP.^8^ The LPPAI ranges from 1 to 10. One study utilized Body Surface Area (BSA) as an outcome measure to compare the included patients’ affected body surface area prior to and after the administration of JAK inhibitors. The remaining studies (13/15) based their reported outcomes based on physician general impressions categorized into complete, partial, or no resolution of the LP-induced lesions.

**Table 1:** Outcomes of JAK Inhibitor Therapy Use for Lichen Planus.

Treatment group (%, n/N)	Study design (n/N)	Treatment outcome (%, n/N)	Mean change in LPPAI measures from baseline (n/N)	Concomitant systemic medications (n/N)	Mean treatment duration, days (n/N)	Recurrence (n/N)	Adverse events (n/N)	Mean follow-up period, months (n/N)
Tofacitinib (50%, 30/60)	Case report (4/9) Retrospective study (2/8) Cohort study (1/8) Case series (1/8)	CR (10%, 3/60)	NR	N (3/3)	180 (1/3)	N (1/3)	Infected hematoma (1/3)	3.5 (3/3)
		PR (60%, 18/30)	−0.53 (7/18)	Y (10/18) N (8/18)	324.4 (16/18)	NR	Creatinine abnormalities (1/18); hemoglobin abnormalities (1/18); hypercholesterolaemia (1/18); hypertriglyceridemia (1/18); weight gain (1/18)	11.9 (15/18)
		NOR (30%, 9/30)	−0.01 (5/9)	Y (8/9) N (1/9)	192 (5/9)	NR	NR	7.2 (5/9)
Baricitinib (26.7%, 16/60)	Case report (4/5) Retrospective study (1/5)	CR (25%, 4/16)	NR	Y (1/4) N (3/4)	135 (4/4)	N (3/4)	Hypercholesterolaemia (1/4)	7.7 (3/4)
		PR (31.3%, 5/16)	−0.7 (5/5)	Y (5/5)	192 (5/5)	NR	Fatigue (1/5); hypercholesterolemia (1/5); neutropenia (1/5); transaminitis (1/5)	NR
		NOR (43.8%, 7/16)	0.01 (7/7)	Y (7/7)	188.5 (7/7)	NR	Hypercholesterolemia (1/7); neutropenia (1/7); transaminitis (1/7)	NR
Ruxolitinib (20%, 12/60)	Cohort study (1/1)	CR (16.7%, 2/12)	NR	N (2/2)	7 (2/2)	NR	Abnormal taste (1/2)	3 (2/2)
		PR (83.3%, 10/12)	NR	N (10/10)	14 (10/10)	NR	NR	3 (10/10)
Upadacitinib (3.3%, 2/60)	Case report (2/2)	CR (100%, 2/2)	NR	N (2/2)	17.5 (2/2)	N (2/2)	NR	9 (2/2)

Abbreviations: CR, Complete Resolution; LPPAI, Lichen Planopilaris Activity Index; N, No; NOR, No Resolution; NR, None Reported; PR, Partial Resolution; Y, Yes.

Additional Details on Lichen Planus Location Distribution, JAK Inhibitor Therapy Dosing, Route of Administration, Concomitant Therapy Use, Baseline BSA and LPPAI, and Time to Achieve LPPAI Changes Are Listed in Supplemental File 2.

### Tofacitinib

Tofacitinib was the most reported JAK inhibitor (50%, 30/60) with 60% (18/30) of patients achieving partial resolution, 30% (9/30) had no resolution, and only 10% (3/30) achieved complete resolution. Sub-analysis of the patients group showed 80% (24/30) of patients tofacitinib on were in the LPP sub-group, with 62.5% (15/24) achieving partial resolution and the other 37.5% (9/24) had no resolution. The LPPAI scores were reported in 8 patients, with 85.7% (6/7) of them achieving a reduction, and only (14.3%) 1/7 experiencing no change in their LPPAI scores. The average reduction in LPPAI score was 0.53 points. Where reported, no patients experienced disease reactivation, defined as worsening disease activity. The average time to reach the reported outcomes was 287.7 days, although reported outcome time was synonymous with treatment duration in 53.3% (16/30) of patients, leading to longer outcome times (Supplemental File 2). Concurrent use of systemic non-JAK inhibitor therapies was noted in 56.7% (17/30) of tofacitinib patients, with dutasteride (52.9%, 9/17) being the most commonly used followed by oral minoxidil and naltrexone (29.4%, 5/17 each). Three patients reported a total of 5 adverse events, including creatinine abnormalities (1/5), an infected hematoma (1/5), hemoglobin abnormalities (1/5), and hypercholesterolemia (1/5), and weight gain (1/5). One of the patients who experienced weight gain discontinued their medication due to the adverse event.

### Baricitinib

In the reported data for the 16 patients who received baricitinib, 31.3% (5/16) achieved partial resolution, 43.8% (7/16) had no resolution, and 25% (4/16) achieved complete resolution. Sub-analysis of the patients group showed 81.3% (13/16) of patients on baricitinib were in the LPP sub-group, with 38.5% (5/13) achieving partial resolution, 53.8% (7/13) had no resolution, and only 7.7% (1/13) achieved complete resolution. In the studies that utilized the LPPAI, 41.7% (5/12) of the patients experienced a reduction in their LPPAI, with the average reduction being a factor of 0.7016. 33.3% (4/12) of the patients experienced no change in their LPPAI scores, while the remaining 25% (3/12) experienced an increase in their LPPAI scores. Among the patients receiving baricitinib, the average time to outcome observed was 38.1 days. In the patients that had seen complete resolution, no reactivation of disease was reported. Concurrent use of systemic non-JAK inhibitor therapies was noted in 81.3% (13/16) of baricitinib patients, with oral minoxidil (69.2%, 9/13) being the most commonly used followed by dutasteride (23.1%, 3/13). Three of the patients included reported a total of 8 adverse events associated with baricitinib use; hypercholesterolemia (37.5%, 3/8), neutropenia (25%, 2/8), transaminitis (25%, 2/8), and fatigue (12.5%, 1/8). Only one patient discontinued their treatment due to adverse events, related to fatigue.

### Ruxolitinib

12 patients from the included studies received ruxolitinib for LP, 16.7% (2/12) of which achieved complete resolution, whereas the remaining 83.3% (10/12) achieved partial resolution. LPPAI scores were not reported for this patient cohort, however, the BSA was measured prior to and after receiving ruxolitinib. A total of 91.6% (11/12) patients achieved a reduction in the BSA affected by LP, with the average reduction being 5.6%. Only one patient did not achieve any net changes in their BSA. The average time to outcome observed was 12.8 days. No concurrent use of systemic non-JAK inhibitor therapies was noted with patients receiving ruxolitinib. One patient reported an adverse event, where the patient experienced abnormal taste due to ruxolitinib therapy. Reactivation data for this patient cohort was not reported.

### Upadacitinib

Finally, the outcomes of 2 patients receiving upadacitinib for LP, both of which achieved complete resolution. Both of the patients did not experience any disease reactivation during the follow-up period, and the time to outcome was 7 days for one patient, and 28 days for the other patient. LPPAI scores and BSA measures for those patients prior to and after the treatment course were not reported. No concurrent use of systemic non-JAK inhibitor therapies was noted with patients receiving upadacitinib. No concurrent Data addressing the presence of adverse events was not reported in the studies examining upadacitinib in treating LP. Given the limited sample size of 2 patients, these reports may be not be sufficient to draw conclusions on the efficacy of upadacitinib in LP.

## Discussion

This systematic review summarizes the current literature findings in terms of the treatment outcomes of JAK inhibitors in patients with LP. A total of 15 studies, with a total of 60 instances of JAK inhibitor therapy across 56 patients (multiple JAK inhibitor use in 4 patients), were included in this review. In the patients with reported data, 73.3% (44/60) of instances with JAK inhibitor use achieved partial or complete resolution, and 26.7% (16/60) achieved no resolution of LP following their course of JAK inhibitors therapy. This data suggests that targeting the JAK pathway can yield effective outcomes in the context of LP pathophysiology. The data compiled in this review also reveals the heterogeneity of LP outcome measures reported in the current literature.

Although the pathophysiology of LP has yet to be clearly identified, current literature suggests that LP is caused by the response of keratinocytes to CD8 +T cell-mediated cytotoxic signalling. The cytotoxic action of CD8 +T cells ultimately induces cellular apoptosis in keratinocytes.^9^ It is thought that the *T* cell-mediated response is further potentiated through the keratinocytes being primed by the IFN-y pathway, as this pathway increases the sensitivity of the keratinocytes to the inflammatory signalling.^10^ Following an increase in IFN-y activity, MHC I expression by keratinocytes is increased primarily through the JAK2/STAT1 pathway.^11^

Molecular studies have demonstrated that inhibiting the JAK2/STAT1 pathway can potentially protect keratinocytes from cytotoxic responses.^12^ IFN-y activity is associated with an increase in the expression of the CXCL10, CXCL9, and CXCL11 chemokines, particularly at the papillary dermis and the dermal-epidermal junction.^13^ These sites are of significance, as they are typically utilized by lymphocytes to access the epidermis. Adding to the validity of the association of the chemokines to the pathophysiology of LP, studies have also uncovered that the expression of CXCR3, a CXCL10 receptor found on lymphocytes, was found to be increased.^13^ These findings further support the hypothesis that the IFN-y/CXCL10 axis can be a viable target in treating LP. In order to propagate its action, IFN-y utilizes JAK as a main signal transducer.^11^ JAK functions through activating STATs, which are phosphorylated following their activation. Once activated, STATs translocate to the nucleus, where they bind to specific gene regulators and initiate the transcription of CXCL10.^13^ However, inhibiting the JAK-STAT pathway through inhibiting JAK action, can cease the effect of IFN-y on the inflammatory processes observed in LP.^11^

Conventionally, LP has been treated with topical corticosteroids which are often effective at limiting the disease’s progression during the period of administration. However, patients often experience disease reactivation once topical corticosteroids are stopped, and thus, exploring other therapeutic agents becomes of significant relevance.^14^ Considering the adverse impacts of corticosteroids in treating LP, the safety profile offered by JAK inhibitors adds to the promising aspect of these medications as therapeutic agents in treating LP. As reported by our results, only 3.3% (2/56) of the patients included reported stopping the JAK inhibitors due to adverse events. Additionally, only 12.5% (7/56) patients reported adverse events.

Apart from topical corticosteroids, several second-line therapies have been approved to treat LP. Second line therapies include systemic glucocorticoids, phototherapy using ultraviolet B light and psoralen plus ultraviolet A light, as well as oral retinoids.^15^ However, similar to topical corticosteroids, the current data from the literature points to either the insufficiency in these agents as sole treatments for LP, or safety concerns with their long-term use. Particularly, long-term usage of systemic glucocorticoids has been implicated in serious side effects such as increases in the risk of developing skin thinning and ecchymoses, as well as hypertension and premature atherosclerotic disease.^16^ On the other hand, the data that supports the therapeutic benefit of utilizing phototherapy and oral retinoids is limited, and has been documented as appropriate mainly for treating cutaneous lichen planus.^17^ Conversely, emerging evidence suggests that JAK inhibitors can potentially be prescribed for additional indications to treat various types of LP, as they selectively target one of the main mechanistic pathways through which the disease develops.^18^ Such findings highlight the necessity of further exploring the utility of JAK inhibitors in such a scope, especially since there are currently no FDA-approved drugs for LP.

The number of participants in each study included varied, which makes it challenging to offer an accurate comparison between the different JAK inhibitors. Furthermore, there were no randomized controlled trials included in the study, which affects the level of evidence included. This also makes it more challenging to provide more generalizable data, as the differences in patient characteristics as well as confounding factors may have not been effectively accounted for. Another limitation in this study is the lack of uniformity in outcome measures utilized. As alluded to previously, the studies varied in their choice of outcome measure used to quantify the utility of JAK inhibitors. Such heterogeneity adds another challenge in comparing the objective effects of the therapeutic agents in treating LP.

Despite the limitations, our review demonstrates a comprehensive summary of the current clinical impacts of utilizing JAK inhibitors to treat LP. The current findings indicate that the majority of the patients experience an improvement in their disease prognosis, with a small minority experiencing adverse events. Notably, 50% (28/56) of the patients included were also receiving concurrent systemic therapies. As such, it would be of further interest to investigate the impact of utilizing systemic therapies as an add-on to JAK inhibitors in comparison to JAK inhibitors as a solitary treatment in LP. Finally, our review’s results highlight the importance of developing and utilizing a uniform outcome measures tool for LP, as this would add to the validity of the results published in literature and allow for published results to be more generalizable.

## Supplemental Material

Figure S1 - Supplemental material for The Use of Janus Kinase Inhibitors for Lichen Planus: An Evidence-Based Review Click here for additional data file. Supplemental material, Figure S1, for The Use of Janus Kinase Inhibitors for Lichen Planus: An Evidence-Based Review by Abrahim Abduelmula, Ahmed Bagit, Asfandyar Mufti, Katie C.Y. Yeung and Jensen Yeung in Journal of Cutaneous Medicine and Surgery

Online supplementary file 1 - Supplemental material for The Use of Janus Kinase Inhibitors for Lichen Planus: An Evidence-Based Review Click here for additional data file. Supplemental material, Online supplementary file 1, for The Use of Janus Kinase Inhibitors for Lichen Planus: An Evidence-Based Review by Abrahim Abduelmula, Ahmed Bagit, Asfandyar Mufti, Katie C.Y. Yeung and Jensen Yeung in Journal of Cutaneous Medicine and Surgery

Online supplementary file 2 - Supplemental material for The Use of Janus Kinase Inhibitors for Lichen Planus: An Evidence-Based Review Click here for additional data file. Supplemental material, Online supplementary file 2, for The Use of Janus Kinase Inhibitors for Lichen Planus: An Evidence-Based Review by Abrahim Abduelmula, Ahmed Bagit, Asfandyar Mufti, Katie C.Y. Yeung and Jensen Yeung in Journal of Cutaneous Medicine and Surgery

Online supplementary file 3 - Supplemental material for The Use of Janus Kinase Inhibitors for Lichen Planus: An Evidence-Based Review Click here for additional data file. Supplemental material, Online supplementary file 3, for The Use of Janus Kinase Inhibitors for Lichen Planus: An Evidence-Based Review by Abrahim Abduelmula, Ahmed Bagit, Asfandyar Mufti, Katie C.Y. Yeung and Jensen Yeung in Journal of Cutaneous Medicine and Surgery
