# Supplementary figures and images for: The Use of Janus Kinase Inhibitors for Lichen Planus: An Evidence-Based Review
Source: J Cutan Med Surg. 2023 Feb 23;27(3):271–6. doi: 10.1177/12034754231156100 (PMC10291104; doi:10.1177/12034754231156100)

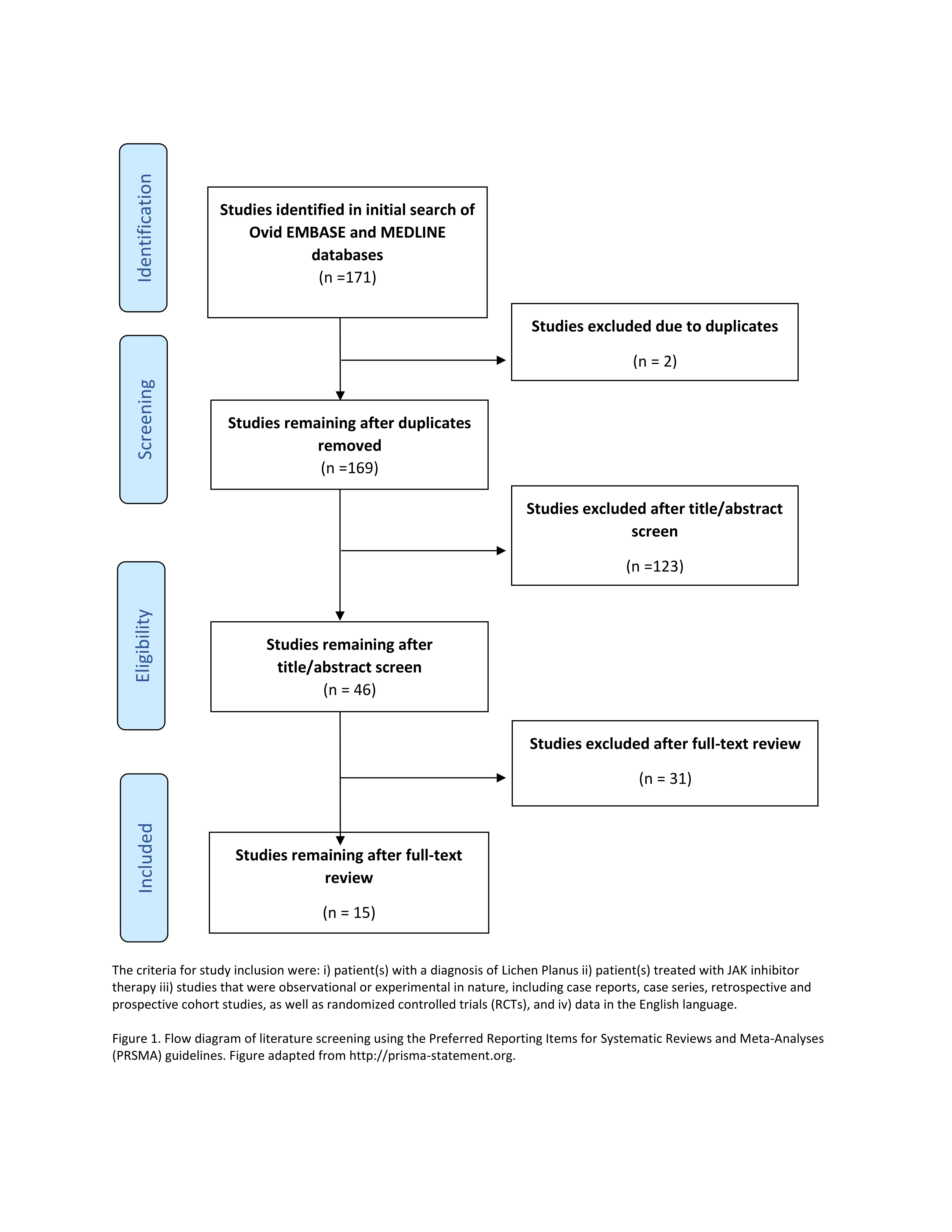

Supplement: Figure S1 - Supplemental material for The Use of Janus Kinase Inhibitors for Lichen Planus: An Evidence-Based Review [file sj-jpg-1-cms-10.1177_12034754231156100.jpg]
