# Supplementary material for: The Use of Janus Kinase Inhibitors for Lichen Planus: An Evidence-Based Review
Source: J Cutan Med Surg. 2023 Feb 23;27(3):271–6. doi: 10.1177/12034754231156100 (PMC10291104; doi:10.1177/12034754231156100)
Supplement: Online supplementary file 1 - Supplemental material for The Use of Janus Kinase Inhibitors for Lichen Planus: An Evidence-Based Review [file sj-docx-1-cms-10.1177_12034754231156100.docx]

**Supplemental File 1.** Search strategy used for literature screening.

Database(s):

Ovid MEDLINE: Epub Ahead of Print, In-Process & Other Non-Indexed Citations, Ovid MEDLINE® Daily and Ovid MEDLINE® 1946-Present, Embase Classic+Embase 1947 to 2022 October 16, 2022

Search strategy:

| **#** | **Searches** | **Results** |
| --- | --- | --- |
| 1 | Lichen*.mp. [mp=ti, ab, hw, tn, ot, dm, mf, dv, kf, fx, dq, nm, ox, px, rx, an, ui, sy] | 70620 |
| 2 | Baricitinib.mp. [mp=ti, ab, hw, tn, ot, dm, mf, dv, kf, fx, dq, nm, ox, px, rx, an, ui, sy] | 4671 |
| 3 | Tofacitinib.mp. [mp=ti, ab, hw, tn, ot, dm, mf, dv, kf, fx, dq, nm, ox, px, rx, an, ui, sy] | 10535 |
| 4 | Ruxolitinib.mp. [mp=ti, ab, hw, tn, ot, dm, mf, dv, kf, fx, dq, nm, ox, px, rx, an, ui, sy] | 10866 |
| 5 | Upadacitinib.mp. [mp=ti, ab, hw, tn, ot, dm, mf, dv, kf, fx, dq, nm, ox, px, rx, an, ui, sy] | 1908 |
| 6 | Fedratinib.mp. [mp=ti, ab, hw, tn, ot, dm, mf, dv, kf, fx, dq, nm, ox, px, rx, an, ui, sy] | 968 |
| 7 | abrocitinib.mp. [mp=ti, ab, hw, tn, ot, dm, mf, dv, kf, fx, dq, nm, ox, px, rx, an, ui, sy] | 438 |
| 8 | Peficitinib.mp. [mp=ti, ab, hw, tn, ot, dm, mf, dv, kf, fx, dq, nm, ox, px, rx, an, ui, sy] | 374 |
| 9 | Delgocitinib.mp. [mp=ti, ab, hw, tn, ot, dm, mf, dv, kf, fx, dq, nm, ox, px, rx, an, ui, sy] | 186 |
| 10 | JAK*.mp. [mp=ti, ab, hw, tn, ot, dm, mf, dv, kf, fx, dq, nm, ox, px, rx, an, ui, sy] | 127864 |
| 11 | 2 or 3 or 4 or 5 or 6 or 7 or 8 or 9 or 10 | 141599 |
| 12 | 1 and 11 | 215 |
| 13 | remove duplicates from 12 | 171 |
