# Supplementary material for: The Use of Janus Kinase Inhibitors for Lichen Planus: An Evidence-Based Review
Source: J Cutan Med Surg. 2023 Feb 23;27(3):271–6. doi: 10.1177/12034754231156100 (PMC10291104; doi:10.1177/12034754231156100)
Supplement: Online supplementary file 2 - Supplemental material for The Use of Janus Kinase Inhibitors for Lichen Planus: An Evidence-Based Review [file sj-docx-2-cms-10.1177_12034754231156100.docx]

**Supplemental File 2**. Cases of Lichen Planus treated with JAK inhibitor therapy.

Abbreviations: AE, adverse event; BID, twice daily; BSA, body surface area; CAS, case series; COH, cohort study; CR, complete resolution; CRS, case report; CS, corticosteroids; F, female; HCQ, hydroxychloroquine; ILES, intralesional; IV, intravenous; LPPAI, Lichen Planopilaris Activity Index M, male; MMF, mycophenolate mofetil; MTX, methotrexate; N, no; NOR, no response; NR, none reported; QD, once daily; PO, oral; PR, partial response; Q1W, every 1 week; RCT, randomized controlled trial; RS, retrospective study; SC, subcutaneous; TOP, topical; Y, yes.

| **Study** | **Study type (level of evidence)** | **Sample size of study extracted** | **Mean age (range), years** | **Sex (n)** | **Comorbidities (n)** | **Mean duration, months** | **Type of LP** | **Location of LP** | **Failed prior therapies [route, dose, and frequency] (n)** | **Concurrent topical therapies [route, dose, and frequency] (n)** | **Concurrent non-biologic systemic therapies [route, dose, and frequency] (n)** | **BSA**  **pre-/post- treatment** | **Pre-LPPAI/Post-LPPAI treatment** | **JAK inhibitors therapy [route, dose, and frequency] (n)** | **Outcomes for JAK inhibitors [CR, PR, or NOR] (n)** | **Resolution period for systemic biologic therapy, days** | **Recurrence despite JAK inhibitor therapy (n)** | **AEs for JAK inhibitor therapy (n)** | **Follow-up period, months** |
| --- | --- | --- | --- | --- | --- | --- | --- | --- | --- | --- | --- | --- | --- | --- | --- | --- | --- | --- | --- |
| Moussa 2022^1^ | RS (2b) | 1 of 12 | 48 | F | NR | 3 | Lichen planopilaris (1) | Forehead (1) | CS [TOP, NR, NR] (1); HCQ [NR, NR, NR] (1) | NR | Dutasteride [NR, 0.5 mg, QD] (1); minoxidil [PO, 0.5-3 mg, QD] (1); spironolactone [NR, 50-75 mg, QD] (1) | NR/NR | 5/1.08 | Baricitinib [PO, 3.4 mg, QD] (1) | PR (1) | 150 | N (1) | Fatigue (1); hypercholesterolemia (1); neutropenia (1); transaminitis (1) | NR |
| Moussa 2022^1^ | RS (2b) | 1 of 12 | 37 | F | NR | 3 | Lichen planopilaris (1) | Scalp (1) | CS [TOP, NR, NR] (1); CS [PO, NR, NR] (1); HCQ [NR, NR, NR] (1) | NR | Bicalutamide [NR, 10 mg, QD] (1); minoxidil [PO, 0.5-3 mg, QD] (1) | NR/NR | 7/7 | Baricitinib [PO, 6.8 mg, QD] (1) | NOR (1) | 180 | NR | Hypercholesterolemia (1); neutropenia (1); transaminitis (1) | NR |
| Moussa 2022^1^ | RS (2b) | 1 of 12 | 26 | F | NR | 18 | Lichen planopilaris (1) | Forehead (1) | Calcineurin inhibitor [TOP, NR, NR] (1); CS [ILES, NR, NR] (1); doxycycline [NR, NR, NR] (1) | NR | Minoxidil [PO, 0.5-3 mg, QD] (1); spironolactone [NR, 50-75 mg, QD] (1) | NR/NR | 4.25/4.25 | Tofacitinib [NR, NR, NR] (1); baricitinib [PO, 6.8 mg, QD] (1) | NOR (1)/NOR (1) | NR/180 | NR/NR | NR/NR | NR |
| Moussa 2022^1^ | RS (2b) | 1 of 12 | 42 | M | NR | 9 | Lichen planopilaris (1) | Scalp (1) | Cyclosporine [NR, NR, NR] (1); CS [PO, NR, NR] (1); MMF [NR, NR, NR] (1) | CS [TOP, NR, NR] (1) | Minoxidil [PO, 0.5-3 mg, QD] (1) | NR/NR | 5.25/5.25 | Tofacitinib [NR, NR, NR] (1); baricitinib [PO, 6.8 mg, QD] (1) | NOR (1)/NOR (1) | NR/180 | NR/NR | NR/NR | NR |
| Moussa 2022^1^ | RS (2b) | 1 of 12 | 41 | M | NR | 26 | Lichen planopilaris (1) | Scalp (1) | CS [ILES, NR, NR] (1); CS [PO, NR, NR] (1); HCQ [NR, NR, NR] (1); minoxidil [PO, NR, NR] (1); MTX [NR, NR, NR] (1) | CS [TOP, NR, NR] (1) | CS [ILES, NR, NR] (1); finasteride [NR, 0.5-1.25 mg, QD] (1) | NR/NR | 6/3.17 | Tofacitinib [NR, NR, NR] (1); baricitinib [PO, 6.8 mg, QD] (1) | NOR (1)/PR (1) | NR/300 | NR/N (1) | NR/NR | NR |
| Moussa 2022^1^ | RS (2b) | 1 of 12 | 44 | M | NR | 5 | Lichen planopilaris (1) | Forehead (1) | Calcineurin inhibitor [TOP, NR, NR] (1); CS [TOP, NR, NR] (1); CS [ILES, NR, NR] (1); HCQ [NR, NR, NR] (1) | NR | Finasteride [NR, 0.5-1.25 mg, QD] (1); minoxidil [PO, 0.5-3 mg, QD] (1) | NR/NR | 6.67/1 | Baricitinib [PO, 3.4 mg, QD] (1) | PR (1) | 150 | N (1) | NR | NR |
| Moussa 2022^1^ | RS (2b) | 1 of 12 | 28 | F | NR | 9 | Lichen planopilaris (1) | Scalp (1) | CS [ILES, NR, NR] (1); CS [PO, NR, NR] (1); HCQ [NR, NR, NR] (1) | CS [TOP, NR, NR] (1) | Minoxidil [PO, 0.5-3 mg, QD] (1) | NR/NR | 4.58/5 | Baricitinib [PO, 3.4 mg, QD] (1) | NOR (1) | 180 | NR | NR | NR |
| Moussa 2022^1^ | RS (2b) | 1 of 12 | 73 | F | NR | 4 | Lichen planopilaris (1) | Scalp (1) | Bicalutamide [NR, NR, NR] (1); CS [ILES, NR, NR] (1); CS [PO, NR, NR] (1); CS [TOP, NR, NR] (1); tildrakizumab [NR, NR, NR] (1) | NR | Minoxidil [PO, 0.5-3 mg, QD] (1) | NR/NR | 7.33/7.33 | Baricitinib [PO, 6.8 mg, QD] (1) | NOR (1) | 180 | NR | NR | NR |
| Moussa 2022^1^ | RS (2b) | 1 of 12 | 56 | F | NR | 2 | Lichen planopilaris (1) | Scalp (1) | Bicalutamide [NR, NR, NR] (1) | CS [TOP, NR, NR] (1) | Minoxidil [PO, 0.5-3 mg, QD] (1) | NR/NR | 5.58/0.67 | Baricitinib [PO, 3.4 mg, QD] (1) | PR (1) | 150 | N (1) | NR | NR |
| Moussa 2022^1^ | RS (2b) | 1 of 12 | 66 | F | NR | 9 | Lichen planopilaris (1) | Forehead (1) | Ciclosporin [NR, NR, NR] (1); CS [PO, NR, NR] (1); doxycycline [NR, NR, NR] (1); HCQ [NR, NR, NR] (1); MTX [NR, NR, NR] (1); tildrakizumab [NR, NR, NR] (1) | NR | Dutasteride [NR, NR, NR] (1); minoxidil [PO, 0.5-3 mg, QD] (1) | NR/NR | 7/7.33 | Tofacitinib [NR, NR, NR] (1); baricitinib [PO, 6.8 mg, QD] (1) | NOR (1)/NOR (1) | NR/180 | NR/NR | NR/NR | NR |
| Moussa 2022^1^ | RS (2b) | 1 of 12 | 53 | F | NR | 13 | Lichen planopilaris (1) | Forehead (1) | Calcineurin inhibitor [TOP, NR, NR] (1); finasteride [NR, NR, NR] (1); HCQ [NR, NR, NR] (1); MTX [NR, NR, NR] (1); spironolactone [NR, NR, NR] (1) | CS [TOP, NR, NR] (1) | Dutasteride [NR, 0.5 mg, QD] (1); minoxidil [PO, 0.5-3 mg, QD] (1) | NR/NR | 4.58/4.58 | Baricitinib [PO, 3.4 mg, QD] (1) | NOR (1) | 300 | NR | NR | NR |
| Moussa 2022^1^ | RS (2b) | 1 of 12 | 28 | F | NR | 5 | Lichen planopilaris (1) | Scalp (1) | Bicalutamide [NR, NR, NR] (1); CS [TOP, NR, NR] (1); spironolactone [NR, NR, NR] (1) | NR | CS [ILES, NR, NR] (1); minoxidil [PO, 0.5-3 mg, QD] (1) | NR/NR | 5.92/2.83 | Baricitinib [PO, 3.4 mg, QD] (1) | PR (1) | 150 | N (1) | NR | NR |
| Moussa 2022 (1)^2^ | CRS (5) | 1 | 63 | F | Chronic alopecia areata (1) | 0.33 | Erosive lichen planus (1) | Oral mucosa (1) | NR | NR | CS [ILES, NR, NR] (1); CS:prednisolone [NR, 12.5 mg, QD] (1) | NR/NR | NR/NR | Baricitinib [PO, 3.4 mg, BID] (1) | CR (1) | 30 | N (1) | Hypercholesterolaemia (1) | 4 |
| Plante 2020^3^ | RS (2b) | 1 of 9 | 61 | F | Erythema dyschromicum (1); rheumatoid arthritis (1); unspecified arthritis (1); vitiligo (1) | 1 | Lichen planopilaris (1) | Scalp (1) | Doxycycline [NR, NR, NR] (1); dutasteride [NR, NR, NR] (1); HCQ [NR, NR, NR] (1); CS [TOP, NR, NR] (1); tacrolimus [TOP, NR, NR] (1) | Pimecrolimus [TOP, NR, NR] (1) | Dutasteride [NR, NR, NR] (1); naltrexone [NR, NR, NR] (1); CS: triamcinolone [ILES, NR, NR] (1) | NR/NR | NR/NR | Tofacitinib [TOP, 2%, BID] (1) | PR (1) | 510 | N (1) | Creatinine abnormalities (1); hemoglobin abnormalities (1); hypercholesterolaemia (1); hypertriglyceridemia (1) | 17 |
| Plante 2020^3^ | RS (2b) | 1 of 9 | 69 | F | NR | 9 | Lichen planopilaris (1) | Scalp (1) | Doxycycline [NR, NR, NR] (1); dutasteride [NR, NR, NR] (1); finasteride [NR, NR, NR] (1); HCQ [NR, NR, NR] (1); minoxidil [NR, NR, NR] (1); pimecrolimus [TOP, NR, NR] (1); CS [TOP, NR, NR] (1); CS: triamcinolone [ILES, NR, NR] (1) | CS [TOP, NR, NR] (1); pimecrolimus [TOP, NR, NR] (1) | Finasteride [NR, NR, NR] (1); minoxidil [NR, NR, NR] (1); CS: triamcinolone [ILES, NR, NR] (1) | NR/NR | NR/NR | Tofacitinib [TOP, 2%, BID] (1) | NOR (1) | 330 | NR | NR | 11 |
| Plante 2020^3^ | RS (2b) | 1 of 9 | 63 | F | NR | 1 | Lichen planopilaris (1) | Forehead (1) | Finasteride [NR, NR, NR] (1); minoxidil [NR, NR, NR] (1); MMF [NR, NR, NR] (1); CS [TOP, NR, NR] (1) | Pimecrolimus [TOP, NR, NR] (1) | Dutasteride [NR, NR, NR] (1); minoxidil [NR, NR, NR] (1); MMF [NR, NR, NR] (1); naltrexone [NR, NR, NR] (1) | NR/NR | NR/NR | Tofacitinib [TOP, 2%, BID] (1) | PR (1) | 300 | N (1) | NR | 10 |
| Plante 2020^3^ | RS (2b) | 1 of 9 | 49 | F | NR | 3 | Lichen planopilaris (1) | Scalp (1) | HCQ [NR, NR, NR] (1); naltrexone [NR, NR, NR] (1); pimecrolimus [TOP, NR, NR] (1); CS [TOP, NR, NR] (1); CS: triamcinolone [ILES, NR, NR] (1) | CS [TOP, NR, NR] (1); pimecrolimus [TOP, NR, NR] (1) | Naltrexone [NR, NR, NR] (1) | NR/NR | NR/NR | Tofacitinib [PO, 5 mg, BID] (1) | PR (1) | 450 | N (1) | NR | 15 |
| Plante 2020^3^ | RS (2b) | 1 of 9 | 63 | F | NR | 2.5 | Lichen planopilaris (1) | Scalp (1) | Dutasteride [NR, NR, NR] (1); finasteride [NR, NR, NR] (1); HCQ [NR, NR, NR] (1); naltrexone [NR, NR, NR] (1); pimecrolimus [TOP, NR, NR] (1); CS [TOP, NR, NR] (1); CS: triamcinolone [ILES, NR, NR] (1) | CS [TOP, NR, NR] (1); pimecrolimus [TOP, NR, NR] (1) | Dutasteride [NR, NR, NR] (1); minoxidil [NR, NR, NR] (1); naltrexone [NR, NR, NR] (1) | NR/NR | NR/NR | Tofacitinib [PO, 5 mg, BID] (1) | NOR (1) | 300 | NR | NR | 10 |
| Plante 2020^3^ | RS (2b) | 1 of 9 | 33 | M | NR | 2 | Lichen planopilaris (1) | Scalp (1) | HCQ [NR, NR, NR] (1); naltrexone [NR, NR, NR] (1); pimecrolimus [TOP, NR, NR] (1); CS [TOP, NR, NR] (1); triamcinolone [ILES, NR, NR] (1) | CS [TOP, NR, NR] (1); pimecrolimus [TOP, NR, NR] (1) | Naltrexone [NR, NR, NR] (1) | NR/NR | NR/NR | Tofacitinib [PO, 5 mg, BID] (1) | PR (1) | 360 | NR | NR | 12 |
| Plante 2020^3^ | RS (2b) | 1 of 9 | 67 | F | NR | 1 | Lichen planopilaris (1) | Forehead (1) | Dutasteride [NR, NR, NR] (1); naltrexone [NR, NR, NR] (1); pimecrolimus [TOP, NR, NR] (1); CS [TOP, NR, NR] (1) | CS [TOP, NR, NR] (1); pimecrolimus [TOP, NR, NR] (1) | Dutasteride [NR, NR, NR] (1); naltrexone [NR, NR, NR] (1) | NR/NR | NR/NR | Tofacitinib [PO, 5 mg, BID] (1) | PR (1) | 210 | N (1) | NR | 7 |
| Plante 2020^3^ | RS (2b) | 1 of 9 | 61 | F | NR | 2.5 | Lichen planopilaris (1) | Scalp (1) | Dutasteride [NR, NR, NR] (1); excimer laser [NR, NR, NR] (1); HCQ [NR, NR, NR] (1); naltrexone [NR, NR, NR] (1); pimecrolimus [TOP, NR, NR] (1); CS: prednisone [PO, NR, NR] (1) | NR | Dutasteride [NR, NR, NR] (1); minoxidil [NR, NR, NR] (1) | NR/NR | NR/NR | Tofacitinib [PO, 11 mg, QD] (1) | PR (1) | 390 | N (1) | NR | 13 |
| Plante 2020^3^ | RS (2b) | 1 of 9 | 59 | F | NR | 6 | Lichen planopilaris (1) | Scalp (1) | Cyclosporine [NR, NR, NR] (1); dutasteride [NR, NR, NR] (1); excimer laser [NR, NR, NR] (1); HCQ [NR, NR, NR] (1); leflunomide [NR, NR, NR] (1); MMF [NR, NR, NR] (1); naltrexone [NR, NR, NR] (1); pimecrolimus [TOP, NR, NR] (1); prednisone [PO, NR, NR] (1); CS [TOP, NR, NR] (1); tacrolimus [TOP, NR, NR] (1); triamcinolone [ILES, NR, NR] (1) | CS [TOP, NR, NR] (1); pimecrolimus [TOP, NR, NR] (1) | Dutasteride [NR, NR, NR] (1); naltrexone [NR, NR, NR] (1) | NR/NR | NR/NR | Tofacitinib [TOP, 2%, BID] (1) | NOR (1) | 30 | NR | NR | 5 |
| Punchera 2022^4^ | CRS (5) | 1 | 60 | F | NR | 1 | Nail lichen planus (1) | Nail (1) | Acitretin [PO, 25 mg, QD] (1); MTX [NR, NR, NR] (1); triamcinolone [IM, 40 mg, Q4W] (1) | NR | NR | NR/NR | NR/NR | Baricitinib [PO, 4mg, QD] (1) | CR (1) | 180 | N (1) | NR | 15 |
| Seiringer 2020^5^ | CRS (5) | 1 | 51 | M | NR | 30 | Lichen planus (1) | Cutaneous (1) | Azathioprine [NR, NR, NR] (1); apremilast [NR, NR, NR] (1); CS [TOP, NR, NR] (1); CS [ILES, NR, NR] (1); cyclosporine [NR, NR, NR] (1); guselkumab [NR, NR, NR] (1); ixekizumab [NR, NR, NR] (1); MTX [NR, NR, NR] (1); MMF [NR, NR, NR] (1); photochemotherapy [NR, NR, NR] (1); CS [PO, NR, NR] (1); retinoids [PO, NR, NR] (1) | NR | NR | NR/NR | NR/NR | Tofacitinib [PO, 5 mg, BID] (1) | PR (1) | NR | NR | NR | NR |
| Yang 2018^6^ | CAS (4) | 1 of 10 | 55 (33-68) | M | NR | 6 | Lichen planopilaris (1) | Scalp (1) | Excimer laser [NR, NR, NR] (1); HCQ [NR, NR, NR] (1); minoxidil [NR, NR, NR] (1); CS: prednisone [PO, NR, NR] (1); CS [TOP, NR, NR] (1); CS: triamcinolone [ILES, NR, NR] (1) | NR | NR | NR/NR | 5.33/1.42 | Tofacitinib [PO, 5 mg, BID] | PR (1) | 300 | NR | NR | 10 |
| Yang 2018^6^ | CAS (4) | 1 of 10 | 55 (33-68) | M | NR | 15 | Lichen planopilaris (1) | Scalp (1) | MMF [NR, NR, NR] (1) | NR | NR | NR/NR | 5.58/1.75 | Tofacitinib [PO, 5 mg, BID] (1) | PR (1) | 510 | NR | NR | 17 |
| Yang 2018^6^ | CAS (4) | 1 of 10 | 55 (33-68) | M | NR | 1 | Lichen planopilaris (1) | Scalp (1) | Doxycycline [NR, NR, NR] (1); excimer laser [NR, NR, NR] (1); finasteride [NR, NR, NR] (1); HCQ [NR, NR, NR] (1); CS: prednisone [NR, NR, NR] (1); CS [TOP, NR, NR] (1); CS: triamcinolone [ILES, NR, NR] (1) | NR | NR | NR/NR | 7.5/7.5 | Tofacitinib [PO, 5 mg, BID] (1) | NOR (1) | 90 | NR | NR | 3 |
| Yang 2018^6^ | CAS (4) | 1 of 10 | 55 (33-68) | F | NR | 2 | Lichen planopilaris (1) | Forehead (1) | Doxycycline [NR, NR, NR] (1); HCQ [NR, NR, NR] (1); CS [TOP, NR, NR] (1); triamcinolone [ILES, NR, NR] (1) | NR | NR | NR/NR | 6.67/4.58 | Tofacitinib [PO, 5 mg, BID] (1) | PR (1) | 270 | NR | NR | 9 |
| Yang 2018^6^ | CAS (4) | 1 of 10 | 55 (33-68) | F | NR | 1 | Lichen planopilaris (1) | Forehead (1) | Doxycycline [NR, NR, NR] (1); excimer laser [NR, NR, NR] (1); finasteride [NR, NR, NR] (1); HCQ [NR, NR, NR] (1); minoxidil [NR, NR, NR] (1); CS [TOP, NR, NR] (1) | NR | HCQ [NR, NR, NR] (1); CS [ILES, NR, NR] (1) | NR/NR | 5.667/0.333 | Tofacitinib [PO, 5 mg, BID] (1) | PR (1) | 180 | NR | NR | 6 |
| Yang 2018^6^ | CAS (4) | 1 of 10 | 55 (33-68) | F | NR | 10 | Lichen planopilaris (1) | Scalp (1) | Finasteride [NR, NR, NR] (1); minocycline [NR, NR, NR] (1); CS [TOP, NR, NR] (1) | Tacrolimus [TOP, NR, NR] (1) | NR | NR/NR | NR/NR | Tofacitinib [PO, 5 mg, BID] (1) | NOR (1) | 210 | NR | NR | 7 |
| Yang 2018^6^ | CAS (4) | 1 of 10 | 55 (33-68) | F | NR | 2 | Lichen planopilaris (1) | Scalp (1) | CS: triamcinolone [ILES, NR, NR] (1) | NR | NR | NR/NR | NR/NR | Tofacitinib [PO, 5 mg, BID] | PR (1) | 570 | NR | Weight gain (1) | 19 |
| Yang 2018^6^ | CAS (4) | 1 of 10 | 55 (33-68) | M | NR | 1 | Lichen planopilaris (1) | Scalp (1) | Doxycycline [NR, NR, NR] (1); finasteride [NR, NR, NR] (1); HCQ [NR, NR, NR] (1); MMF [NR, NR, NR] (1); CS [TOP, NR, NR] (1) | NR | HCQ [NR, NR, NR] (1) | NR/NR | 6.67/2.08 | Tofacitinib [PO, 5 mg, BID] (1) | PR (1) | 480 | NR | NR | 16 |
| Yang 2018^6^ | CAS (4) | 1 of 10 | 55 (33-68) | F | NR | 1 | Lichen planopilaris (1) | Scalp (1) | Doxycycline [NR, NR, NR] (1); finasteride [NR, NR, NR] (1); CS: triamcinolone [ILES, NR, NR] (1) | NR | NR | NR/NR | 6/4.25 | Tofacitinib [PO, 5 mg, BID] (1) | PR (1) | 300 | NR | NR | 10 |
| Yang 2018^6^ | CAS (4) | 1 of 10 | 55 (33-68) | F | NR | 2 | Lichen planopilaris (1) | Scalp (1) | CS [TOP, NR, NR] (1); doxycycline [NR, NR, NR] (1) | NR | NR | NR/NR | 6.33/2.75 | Tofacitinib [PO, 5 mg, BID] (1) | PR (1) | 60 | NR | NR | 2 |
| Balestri 2022^7^ | CRS (5) | 1 | 45 | F | Chronic psoriatic peripheral arthritis (1); recurrent anterior uveitis (1) | 0.058 | Erosive lichen planus (1) | Oral mucosa (1) | NR | NR | NR | NR/NR | NR/NR | Upadacitinib [NR, 15 mg, QD] (1) | CR (1) | 7 | N (1) | NR | 12 |
| Batra 2020^8^ | CRS (5) | 1 | 27 | M | NR | 4 | Lichen planopilaris (1) | Scalp (1) | Acitretin [NR, NR, NR] (1); clobetasol [TOP, 0.05%, BID] (1); HCQ [PO, NR, NR] (1); minoxidil [TOP, 5%, BID] (1); MMF [NR, NR, NR] (1); naltrexone [NR, 4.5 mg, QD] (1); CS: triamcinolone [IM, 10 mg/L, Q4W] (1) | NR | Dapsone [PO, NR, NR] (1) | NR/NR | NR/NR | Tofacitinib [PO, 5 mg, BID] (1) | PR (1) | 120 | NR | NR | NR |
| Damsky 2020^9^ | CoH (2b) | 1 of 3 | 78 | M | NR | 0.5 | Erosive lichen planus (1) | Oral mucosa (1); ocular (1) | CS: prednisone [PO, 60 mg, QD] (1) | NR | MTX [NR, NR, QD] (1); CS:prednisone [NR, 10 mg, QD] (1) | NR/NR | NR/NR | Tofacitinib [PO, 5 mg, BID] (1) | PR (1) | NR | NR | NR | 15 |
| Damsky 2020^9^ | CoH (2b) | 1 of 3 | 57 | F | NR | 22 | Erosive lichen planus (1) | Oral mucosa (1); esophageal (1) | Cyclosporine [NR, NR, NR] (1); MTX [NR, NR, NR] (1); MMF [NR, NR, NR] (1) | NR | NR | NR/NR | NR/NR | Tofacitinib [PO, 5 mg, BID] (1) | CR (1) | NR | NR | NR | 5 |
| Damsky 2020^9^ | CoH (2b) | 1 of 3 | 67 | F | NR | 6 | Erosive lichen planus (1) | Oral mucosa (1) | Acitretin [NR, NR, NR] (1); prednisone [PO, NR, NR] (1) | NR | NR | NR/NR | NR/NR | Tofacitinib [PO, 5 mg, BID] (1) | CR (1) | NR | NR | NR | 2.5 |
| Iorizzo 2021^10^ | CRS (5) | 1 | 57 | F | Hypothyroidism (1); rheumatoid arthritis (1); alopecia universalis (1) | 2 | Nail lichen planus (1) | Nail (1) | Alitretinoin [NR, 30 mg, QD] (1); MTX [NR, 15 mg, Q1W] (1); MTX [NR, 30 mg, Q1W] (1); CS:triamcinolone acetonide [ILES, 10 mg/mL, Q4W] (1) | NR | NR | NR/NR | NR/NR | Tofacitinib [NR, 5 mg, BID] (1) | PR (1) | 180 | N (1) | NR | NR |
| Kooybaran 2021^11^ | CRS (5) | 1 | 59 | F | Exfoliative esophagitis (1) | 5 | Erosive lichen planus (1) | Oral mucosa (1); esophageal (1) | CS [TOP, NR, NR] (1); dexamethasone [NR, NR, NR] (1); HCQ [NR, NR, NR] (1) | NR | NR | NR/NR | NR/NR | Upadacitinib [NR, 15 mg, QD] (1) | CR (1) | 28 | N (1) | NR | 6 |
| Brumfiel 2022^12^ | CoH (2b) | 1 of 12 | 67 | M | NR | 0.6 | Lichen planus (1) | Cutaneous (1) | CS [TOP, NR, NR] (1) | NR | NR | 6.1% (0.1-26)/0.9% (0.0-6.9) | NR/NR | Ruxolitinib [TOP, 1.5%, BID] (1) | CR (1) | 7 | NR | Abnormal taste (1) | 3 |
| Brumfiel 2022^12^ | CoH (2b) | 1 of 12 | 69 | F | NR | 1.75 | Lichen planus (1) | Cutaneous (1) | Acitretin [NR, NR, NR] (1); CS [TOP, NR, NR] (1); CS [ILES, NR, NR] (1); CS [PO, NR, NR] (1); HCQ [NR, NR, NR] (1) | NR | NR | 6.1% (0.1-26)/0.9% (0.0-6.9) | NR/NR | Ruxolitinib [TOP, 1.5%, BID] (1) | PR (1) | 14 | NR | NR | 3 |
| Brumfiel 2022^12^ | CoH (2b) | 1 of 12 | 81 | F | NR | 2 | Lichen planus (1) | Cutaneous (1) | Calcineurin inhibitor [TOP, NR, NR] (1); CS [TOP, NR, NR] (1); MTX [PO, NR, NR] (1) | NR | NR | 6.1% (0.1-26)/0.9% (0.0-6.9) | NR/NR | Ruxolitinib [TOP, 1.5%, BID] (1) | PR (1) | 14 | NR | NR | 3 |
| Brumfiel 2022^12^ | CoH (2b) | 1 of 12 | 48 | F | NR | 0.5 | Lichen planus (1) | Cutaneous (1) | Acitretin [NR, NR, NR] (1); CS [TOP, NR, NR] (1); CS [PO, NR, NR] (1) | NR | NR | 6.1% (0.1-26)/0.9% (0.0-6.9) | NR/NR | Ruxolitinib [TOP, 1.5%, BID] (1) | PR (1) | 7 | NR | NR | 3 |
| Brumfiel 2022^12^ | CoH (2b) | 1 of 12 | 46 | F | NR | 0.75 | Lichen planus (1) | Cutaneous (1) | CS [TOP, NR, NR] (1) | NR | NR | 6.1% (0.1-26)/0.9% (0.0-6.9) | NR/NR | Ruxolitinib [TOP, 1.5%, BID] (1) | PR (1) | 21 | NR | NR | 3 |
| Brumfiel 2022^12^ | CoH (2b) | 1 of 12 | 56 | M | NR | 0.5 | Lichen planus (1) | Cutaneous (1) | Calcineurin inhibitor [TOP, NR, NR] (1); CS [TOP, NR, NR] (1) | NR | NR | 6.1% (0.1-26)/0.9% (0.0-6.9) | NR/NR | Ruxolitinib [TOP, 1.5%, BID] (1) | PR (1) | 21 | NR | NR | 3 |
| Brumfiel 2022^12^ | CoH (2b) | 1 of 12 | 72 | F | NR | 4 | Lichen planus (1) | Cutaneous (1) | Calcineurin inhibitor [TOP, NR, NR] (1); CS [TOP, NR, NR] (1) | NR | NR | 6.1% (0.1-26)/0.9% (0.0-6.9) | NR/NR | Ruxolitinib [TOP, 1.5%, BID] (1) | PR (1) | 7 | NR | NR | 3 |
| Brumfiel 2022^12^ | CoH (2b) | 1 of 12 | 78 | F | NR | 1 | Lichen planus (1) | Cutaneous (1) | CS [TOP, NR, NR] (1) | NR | NR | 6.1% (0.1-26)/0.9% (0.0-6.9) | NR/NR | Ruxolitinib [TOP, 1.5%, BID] (1) | PR (1) | 14 | NR | NR | 3 |
| Brumfiel 2022^12^ | CoH (2b) | 1 of 12 | 59 | F | NR | 0.3 | Lichen planus (1) | Cutaneous (1) | CS [TOP, NR, NR] (1) | NR | NR | 6.1% (0.1-26)/0.9% (0.0-6.9) | NR/NR | Ruxolitinib [TOP, 1.5%, BID] (1) | PR (1) | 14 | NR | NR | 3 |
| Brumfiel 2022^12^ | CoH (2b) | 1 of 12 | 55 | F | NR | 0.5 | Lichen planus (1) | Cutaneous (1) | CS [TOP, NR, NR] (1) | NR | NR | 6.1% (0.1-26)/0.9% (0.0-6.9) | NR/NR | Ruxolitinib [TOP, 1.5%, BID] (1) | PR (1) | 14 | NR | NR | 3 |
| Brumfiel 2022^12^ | CoH (2b) | 1 of 12 | 34 | F | NR | 0.5 | Lichen planus (1) | Cutaneous (1) | CS [TOP, NR, NR] (1) | NR | NR | 6.1% (0.1-26)/0.9% (0.0-6.9) | NR/NR | Ruxolitinib [TOP, 1.5%, BID] (1) | CR (1) | 7 | NR | NR | 3 |
| Brumfiel 2022^12^ | CoH (2b) | 1 of 12 | 68 | F | NR | 2.6 | Lichen planus (1) | Cutaneous (1) | CS [TOP, NR, NR] (1); CS [PO, NR, NR] (1) | NR | NR | 6.1% (0.1-26)/0.9% (0.0-6.9) | NR/NR | Ruxolitinib [TOP, 1.5%, BID] (1) | PR (1) | 14 | NR | NR | 3 |
| Moussa 2022 (2)^13^ | CRS (5) | 1 | 36 | M | NR | 17 | Lichen planus pemphigoides (1) | Cutaneous (1) | Azathioprine [NR, NR, NR] (1); CS [TOP, NR, NR] (1); CS [PO, NR, NR] (1); cyclosporine [NR, NR, NR] (1); doxycycline [NR, NR, NR] (1); MTX [PO, NR, NR]; phototherapy [NR, NR, NR] | NR | NR | NR/NR | NR/NR | Baricitinib [PO, 3.4 mg, QD] (1) | CR (1) | 180 | NR | NR | NR |
| Li 2022^14^ | CRS (5) | 1 | 28 | M | NR | 0.5 | Lichen planopilaris (1) | Scalp (1) | HCQ [PO, 10 mg, BID] (1) | NR | NR | NR/NR | NR/NR | Baricitinib [PO, 4 mg, QD] (1) | CR (1) | 150 | N (1) | NR | 4 |
| Kooybaran 2022^15^ | CRS (5) | 1 | 77 | F | NR | 40 | Erosive lichen planus (1) | Esophageal (1) | CS [PO, NR, NR] (1) | NR | NR | NR/NR | NR/NR | Tofacitinib [PO, 5 mg, BID] (1) | CR (1) | 180 | N (1) | Infected hematoma (1) | 3 |

References:

| 1. | Moussa A, Bhoyrul B, Asfour L, Kazmi A, Eisman S, Sinclair RD. Treatment of lichen planopilaris with baricitinib: A retrospective study. *J Am Acad Dermatol*. 2022;87(3):663-666. doi:10.1016/j.jaad.2022.02.027 |
| --- | --- |
|  |  |
| 2. | Moussa A, Colla T, Morrison B, Sinclair R. Effective treatment of oral lichen planus with the JAK inhibitor baricitinib. *Australas J Dermatol*. 2022;63(2):276-277. doi:10.1111/ajd.13811 |
|  |  |
| 3. | Plante J, Eason C, Snyder A, Elston D. Tofacitinib in the treatment of lichen planopilaris: A retrospective review. *J Am Acad Dermatol*. 2020;83(5):1487-1489. doi:10.1016/j.jaad.2020.05.104 |
|  |  |
| 4. | Pünchera J, Laffitte E. Treatment of severe nail lichen planus with baricitinib. *JAMA Dermatol*. 2022;158(1):107-108. doi:10.1001/jamadermatol.2021.5082 |
|  |  |
| 5. | Seiringer P, Lauffer F, Pilz AC, Boehmer D, Biedermann T, Eyerich K. Tofacitinib in hypertrophic lichen planus. *Acta Derm Venereol*. 2020;100(14):adv00220. doi:10.2340/00015555-3585 |
|  |  |
| 6. | Yang CC, Khanna T, Sallee B, Christiano AM, Bordone LA. Tofacitinib for the treatment of lichen planopilaris: A case series. *Dermatol Ther*. 2018;31(6):e12656. doi:10.1111/dth.12656 |
|  |  |
| 7. | Balestri R, Bortolotti R, Rech G, Girardelli CR, Zorzi MG, Magnano M. Treatment of oral erosive lichen planus with upadacitinib. *JAMA Dermatol*. 2022;158(4):457-458. doi:10.1001/jamadermatol.2022.0147 |
|  |  |
| 8. | Batra P, Sukhdeo K, Shapiro J. Hair loss in lichen planopilaris and frontal fibrosing alopecia: Not always irreversible. *Skin Appendage Disord*. 2020;6(2):125-129. doi:10.1159/000505439 |
|  |  |
| 9. | Damsky W, Wang A, Olamiju B, Peterson D, Galan A, King B. Treatment of severe lichen planus with the JAK inhibitor tofacitinib. *J Allergy Clin Immunol*. 2020;145(6):1708-1710.e2. doi:10.1016/j.jaci.2020.01.031 |
|  |  |
| 10. | Iorizzo M, Haneke E. Tofacitinib as treatment for nail lichen planus associated with alopecia universalis. *JAMA Dermatol*. 2021;157(3):352-353. doi:10.1001/jamadermatol.2020.4555 |
|  |  |
| 11. | Kooybaran NR, Petzold G, Ströbel P, Schön MP, Mössner R. Alleviation of erosive oral and esophageal lichen planus by the JAK1 inhibitor upadacitinib. *J Dtsch Dermatol Ges*. 2021;19(12):1778-1780. doi:10.1111/ddg.14612 |
|  |  |
| 12. | Brumfiel CM, Patel MH, Severson KJ, et al. Ruxolitinib cream in the treatment of cutaneous lichen planus: A prospective, open-label study. *J Invest Dermatol*. 2022;142(8):2109-2116.e4. doi:10.1016/j.jid.2022.01.015 |
|  |  |
| 13. | Moussa A, Colla TG, Asfour L, Bhoyrul B, Sinclair RD. Effective treatment of refractory lichen planus pemphigoides with a Janus kinase-1/2 inhibitor. *Clin Exp Dermatol*. Published online 2022. doi:10.1111/ced.15344 |
|  |  |
| 14. | Li Z, Yin M, Xu N, Dou X. Baricitinib as an effective treatment for lichen planopilaris. *Dermatol Ther*. Published online 2022:e15882. doi:10.1111/dth.15882 |
|  |  |
| 15. | Kooybaran NR, Petzold G, Schön MP, Mössner R. Esophageal lichen planus successfully treated with the JAK1/3 inhibitor tofacitinib. *J Dtsch Dermatol Ges*. 2022;20(6):858-860. doi:10.1111/ddg.14762 |
|  |  |
