# Supplementary material for: The Use of Janus Kinase Inhibitors for Lichen Planus: An Evidence-Based Review
Source: J Cutan Med Surg. 2023 Feb 23;27(3):271–6. doi: 10.1177/12034754231156100 (PMC10291104; doi:10.1177/12034754231156100)
Supplement: Online supplementary file 3 - Supplemental material for The Use of Janus Kinase Inhibitors for Lichen Planus: An Evidence-Based Review [file sj-docx-3-cms-10.1177_12034754231156100.docx]

**Supplemental File 3: Summary of Characteristics and Clinical Outcomes of LP in Patients on JAK Inhibitors.**

| **Demographics** |  |
| --- | --- |
| Age (years): Mean Age + Std Dev | 54.5 + 13.8 |
| Male (n, %) | 15 (26.8) |
| Female (n, %) | 41 (73.2) |
| **JAK Inhibitor (n, %)** | |
| Tofacitinib | 30 (50) |
| Baricitinib | 16 (26.7) |
| Ruxolitinib | 12 (20) |
| Upadacitinib | 2 (3.3) |
| **Distribution of LP (n, %)** |  |
| Scalp | 24 (42.9) |
| Forehead | 14 (25) |
| Cutaneous | 12 (22.2) |
| Oral mucosa | 6 (10.7) |
| Nail | 2 (3.6) |
| Esophageal | 2 (3.6) |
| Ocular | 1 (1.8) |
| **Concurrent systemic therapies** |  |
| Minoxidil | 15 (25) |
| Dutasteride | 9 (15) |
| Intralesional corticosteroids | 6 (10) |
| Naltrexone | 6 (10) |
| Finasteride | 3 (5) |
| Oral corticosteroids | 2 (3.3) |
| **Previous treatments for LP (n, %)** |  |
| Topical Corticosteroids | 35 (62.5) |
| Hydroxychloroquine | 23 (41.1) |
| Calcineurin Inhibitors | 15 (26.8) |
| Oral corticosteroids | 12 (21.4) |
| Intralesional corticosteroids | 12 (21.4) |
| Doxycycline | 11 (19.6) |
| Finasteride | 9 (16.1) |
| Methotrexate | 9 (16.1) |
| Mycophenolate mofetil | 8 (14.3) |
| Naltrexone | 7 (12.5) |
| Retinoids | 7 (12.5) |
| Biologics | 6 (10.7) |
| Dutasteride | 6 (10.7) |
| Minoxidil | 6 (10.7) |
| Bicalutamide | 3 (5.4) |
| Phototherapy | 3 (5.4) |
| **Drug Discontinuation (n, %)** |  |
| Yes | 2 (3.3) |
| No | 5 (8.3) |
| NR | 53 (88.3) |
| **Clinical Outcomes (n, %)** | |
| CR | 11 (18.3) |
| PR | 33 (55) |
| NOR | 16 (26.7) |
| **Resolution Period (Days)** |  |
| Mean Duration | 177.1 |
| Duration Range | 7 - 570 |
|  | |

*CR: Complete resolution, PR: Partial resolution, NR: not reported, n: number of participants, %: percentages*
